# Extradural Dermoid Cyst of the Cerebellar Convexity in an Elderly Patient: A Rare Case

**DOI:** 10.7759/cureus.116471

**Published:** 2026-09-18

**Authors:** Aziz Bedioui, Mohamed Zouaghi, Ines Chelly, Ghassen Gader, Kamel Bahri

**Affiliations:** 1 Department of Neurosurgery, Center for Traumatology and Major Burns, Ben Arous, TUN; 2 Department of Neurosurgery, Faculty of Medicine of Tunis, Tunis, TUN; 3 Department of Pathology, Faculty of Medicine of Tunis, Tunis, TUN

**Keywords:** bone erosion, brain tumour, dermoid cyst, extradural, neurosurgery, posterior fossa, retrosigmoid approach

## Abstract

Dermoid cysts (DCs) are rare tumours originating from ectodermal and mesodermal tissues. They usually occur in the midline, especially in the posterior fossa. In this study, we describe an interesting case of a 73-year-old patient who presented with mild headaches, an imbalanced gait, and a slow, progressive hearing loss in the left ear. A brain CT scan showed a hypodense left cerebellar convexity extra-axial mass, containing some calcifications and causing bone erosion. On MRI, this tumour had a low T1 signal, a heterogeneous T2 signal, and a heterogeneous signal on diffusion-weighted images, with discrete peripheral enhancement. The patient was operated on through a left retrosigmoid approach, during which a yellowish, soft, waxy extra-dural mass was removed. Post-operative follow-up was uneventful. Histological study found stratified keratinized tissue, and tumour walls were covered with Malpighian epithelium. These tumours usually have slow, progressive symptoms depending on location; however, severe, life-threatening presentations are possible. CT scan and MRI are useful to establish the diagnosis of dermoid cysts. Maximal safe resection is the treatment of choice.

## Introduction

Intracranial dermoid cyst (DC) is a rare tumour, representing around 0.04-0.7% of all intracranial lesions, occurring usually in the posterior fossa and midline [1-4]. It is a benign tumour deriving from both ectodermal and mesodermal structures [1,2].

Extradural intracranial DC is an extremely rare occurrence [5,6], and to our knowledge, only 25 cases have been reported in the literature so far. Additionally, these tumours are commonly discovered among children and young adults, given their congenital nature [1,7-9]. Furthermore, extradural intracranial DCs located in the cerebellar convexity have only been reported in two previous cases [1].

We present a new rare case of an extradural DC discovered in an elderly patient, highlight its radiological and histological features, and note relevant characteristics.

## Case presentation

A 73-year-old man with a history of type 2 diabetes, hypertension, and coronary disease presented with mild headaches associated with an imbalanced gait and a slow, progressive hearing loss in the left ear, both appearing two years prior. Physical examination found a soft tissue mass behind the left ear without skin abnormalities. Neurological exam found a static and left kinetic cerebellar syndrome, left hypoacusis, and a horizontal nystagmus.

A brain computed tomography (CT) scan showed an extra-axial voluminous hypodense mass, containing some intratumoral calcifications, in the left cerebellar convexity, with bone erosion (Figure 1). Brain magnetic resonance imaging (MRI) showed a lesion measuring 75 x 69 x 72 mm. It had a hypersignal on T1-weighted imaging (WI) and a heterogeneous hypersignal on T2-WI. This tumour presented with irregular peripheral enhancement. In diffusion WI, tumour signal was heterogeneous. Angio-MR showed inner displacement of the ipsilateral transverse and sigmoid sinuses with compromised venous drainage. This tumour compressed and displaced the left cerebellar hemisphere, as well as the fourth ventricle; a mild hydrocephalus was observed upstream. It also pushed the left cerebellar tentorium and the occipital lobe upwards. Figure 2 summarizes MRI aspects of the tumour.

**Figure 1:**
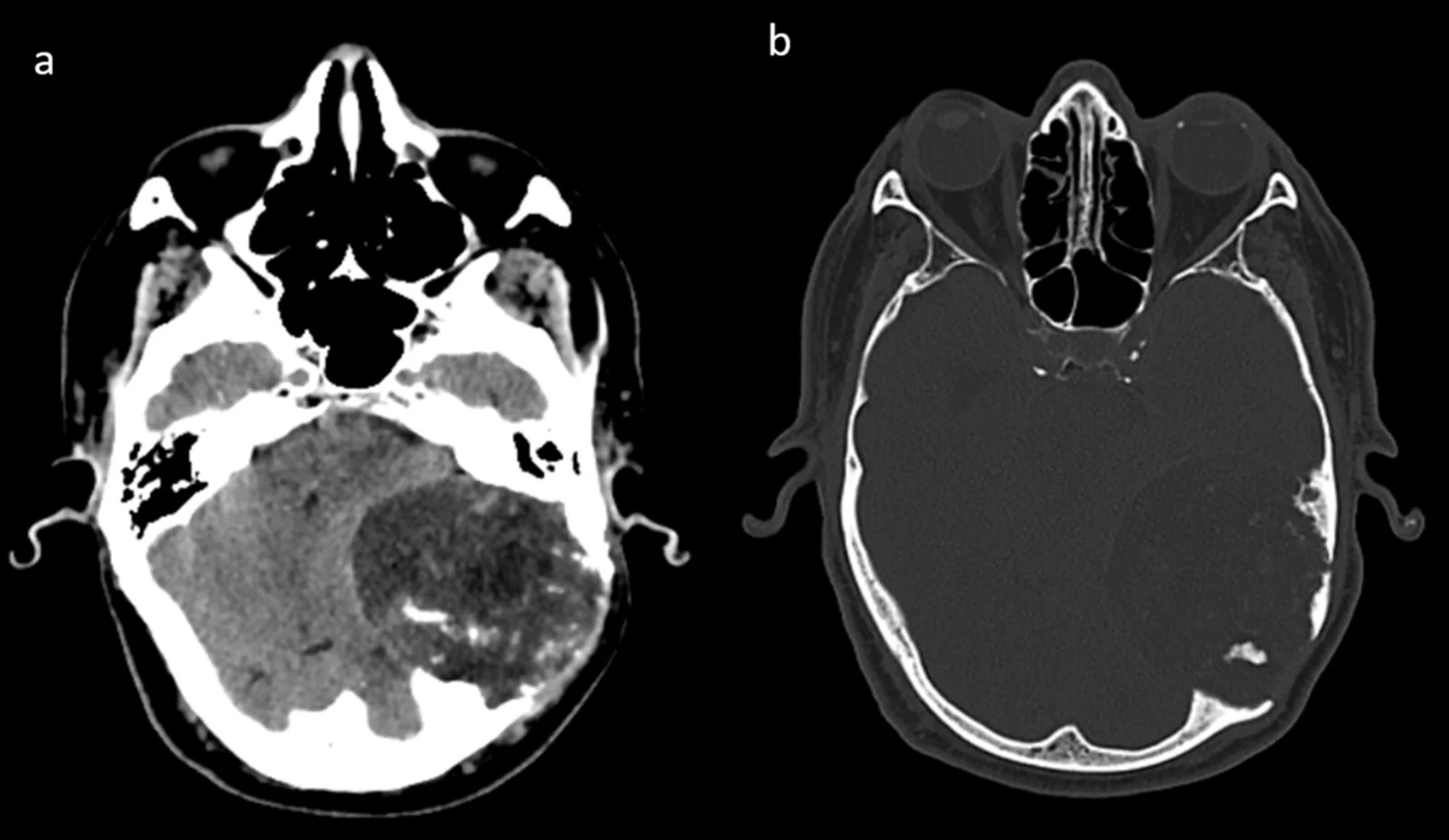
Brain computed tomography scan showing the extradural left cerebellar tumour The parenchymal window (a) shows the extra-axial, voluminous hypodense mass, containing some intratumoral calcifications, in the left cerebellar convexity, with mass effect on the ipsilateral cerebellar hemisphere and brainstem. Bone window (b) shows bone erosion next to the tumour.

**Figure 2:**
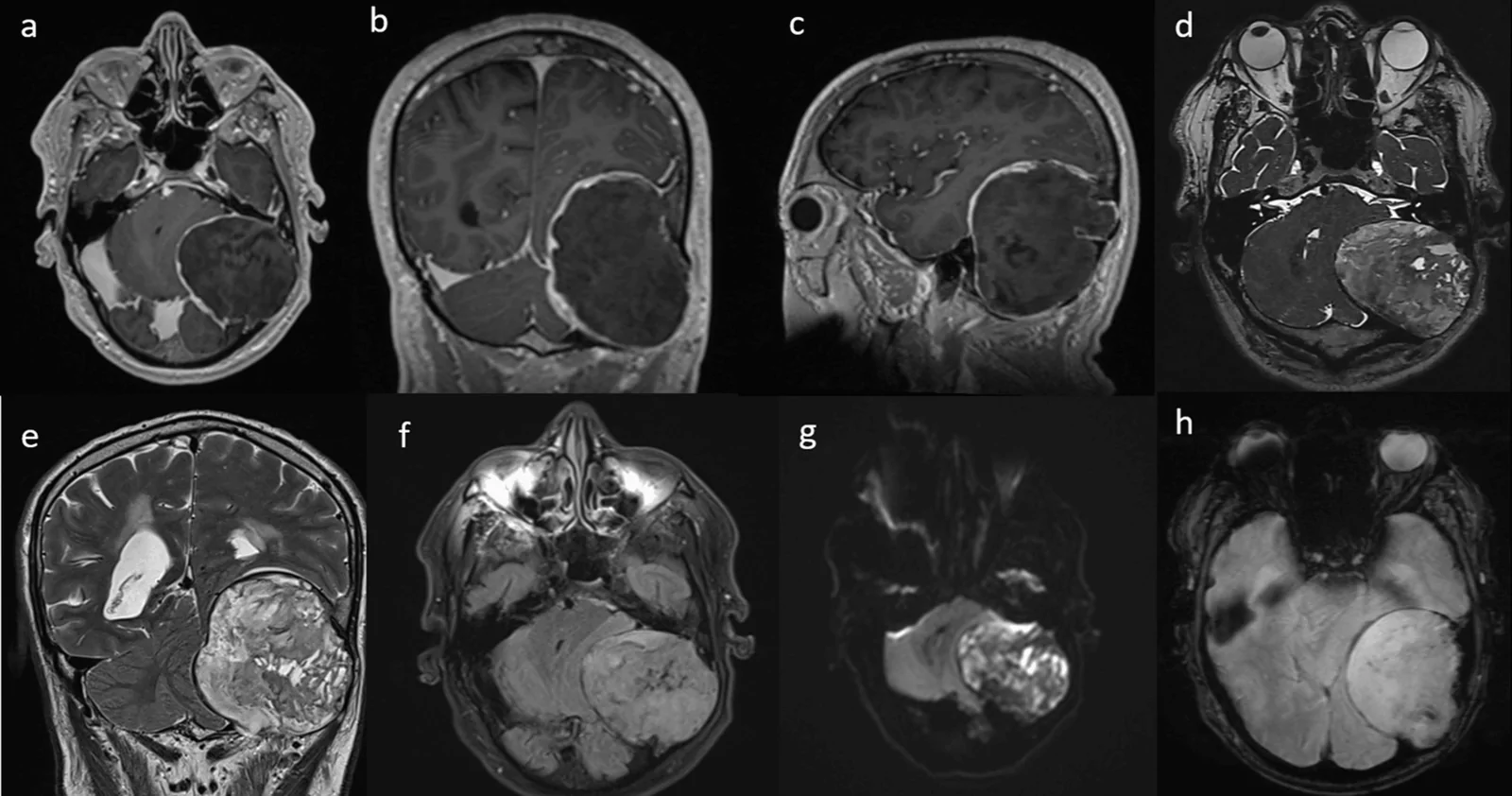
Brain magnetic resonance imaging showing the extradural left cerebellar tumour The lesion had a hyposignal on T1 weighted imaging (WI) with, an irregular peripheral enhancement (a, b, c). It has a heterogeneous hypersignal on T2-WI (d, e). Fluid-attenuated inversion recovery (FLAIR) images (f) show intratumoral hyposignal and the absence of oedema in the cerebellum, brain stem, and occipital lobe. In diffusion WI (g), tumour signal is heterogeneous. Axial, coronal, and sagittal images (a, b, c) show inner displacement of the ipsilateral transverse and sigmoid sinus, as well as upper displacement of the tentorium and the occipital lobe. Susceptibility WI (h) showed intratumoral hyposignal corresponding to calcifications.

The patient was operated on through a left retrosigmoid approach. We found a yellowish, soft, waxy extra-dural mass, well delineated from the dura (Figure 3). The lateral sinus was deviated inward along with the dura. A piece-meal gross total resection (GTR) was performed. The dura was intact. The eroded bone was removed, followed by a cranioplasty with bone cement.

**Figure 3:**
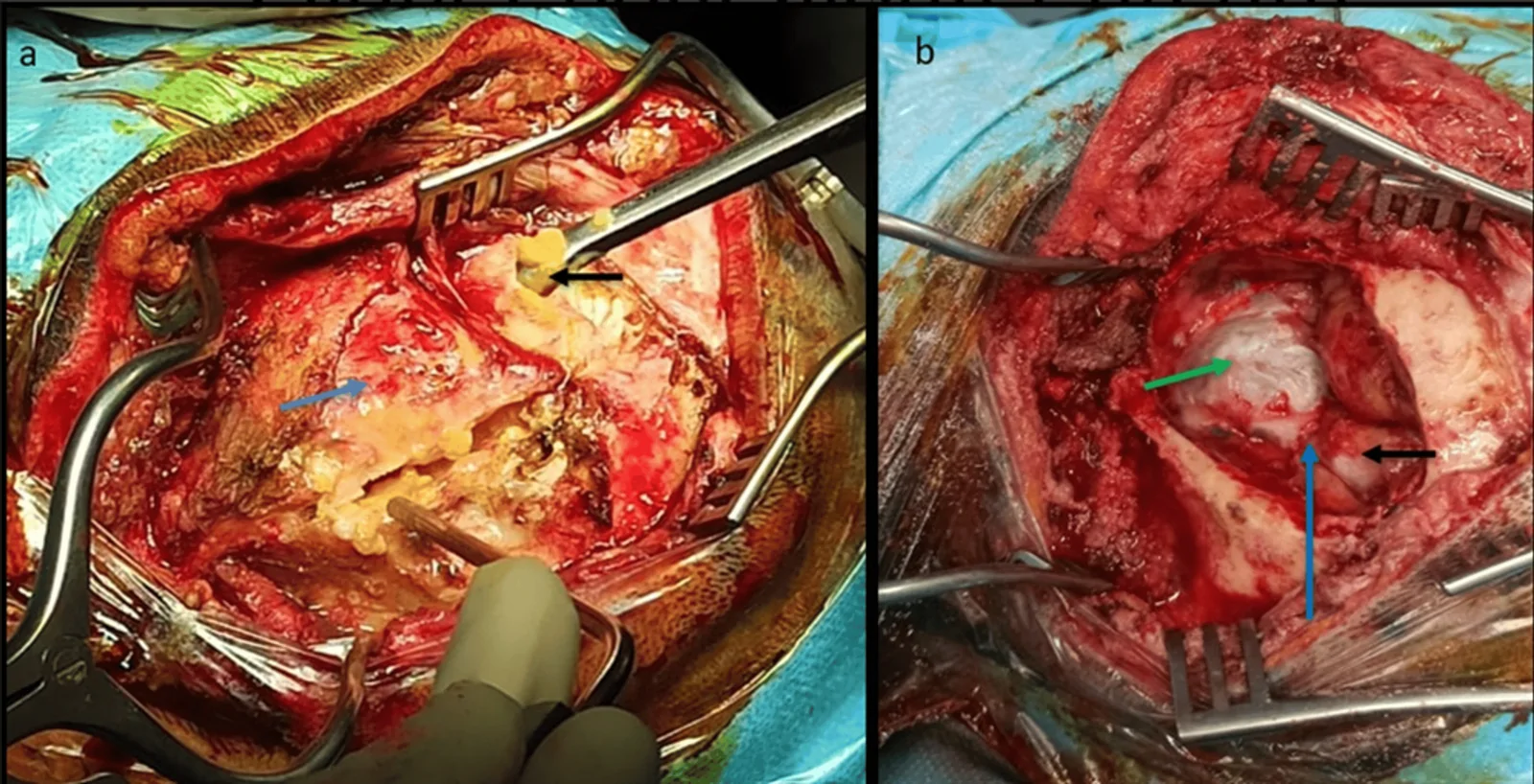
Perioperative photographs of the tumour The left image (a) shows the yellowish, waxy tumour content removed with forceps (black arrow) and the external cyst capsule (blue arrow). The right image (b) was taken after the dermoid cyst removal; it shows the complete resection. The underlying cerebellar dura (green arrow) and occipital dura (black arrow), as the deviated transverse sinus (blue arrow) are shown.

Histological study found stratified keratinized tissue, and tumour walls were covered with Malpighian epithelium (Figure 4). The diagnosis of an extradural dermoid cyst was established.

**Figure 4:**
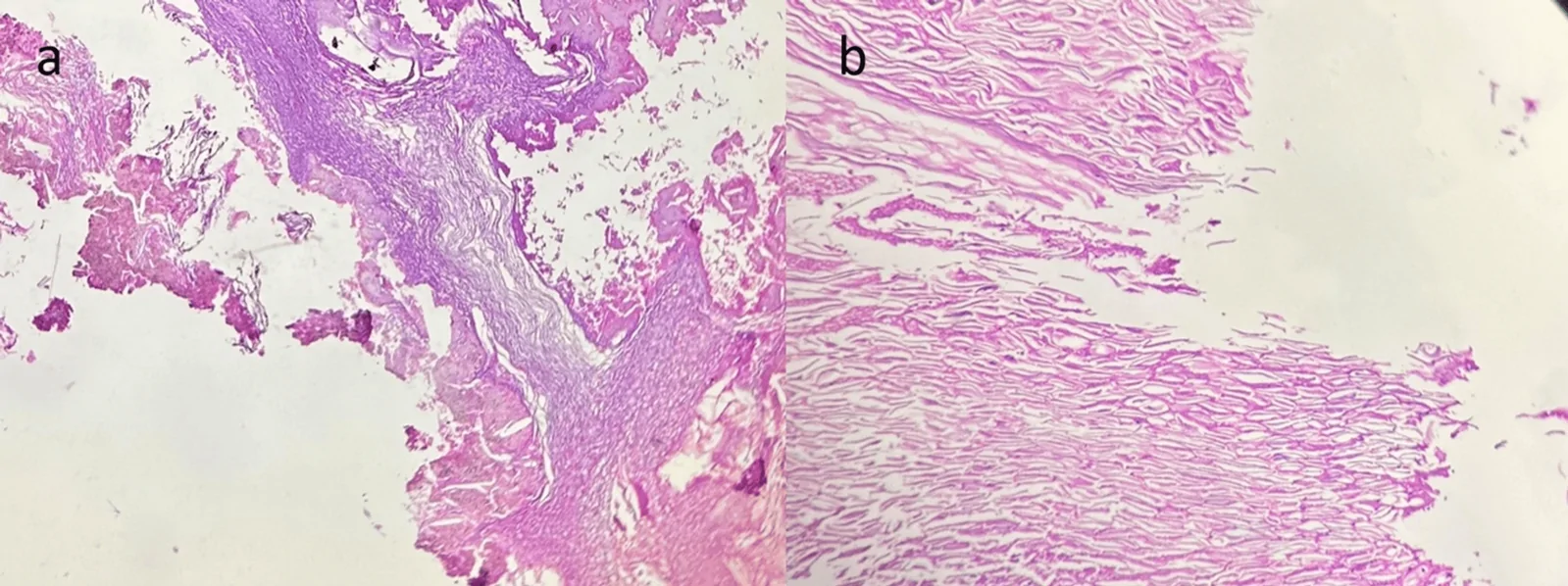
Microscopical images of the histological study (a) Hematoxylin and eosin staining x200 magnification. b) Hematoxylin and eosin staining x400 magnification, both showing a stratified keratinized tissue, and tumour walls covered with Malpighian epithelium, with the absence of atypical cells.

The postoperative course was uneventful. A Day 1 postoperative CT scan showed complete removal of the tumour without complications (Figure 5). The patient was discharged three days after surgery. Subsequent follow-up was uneventful. At one-year follow-up, the patient showed remarkable improvement in gait imbalance, while hearing loss was stable. One-year postoperative MRI showed no signs of recurrence (Figure 6).

**Figure 5:**
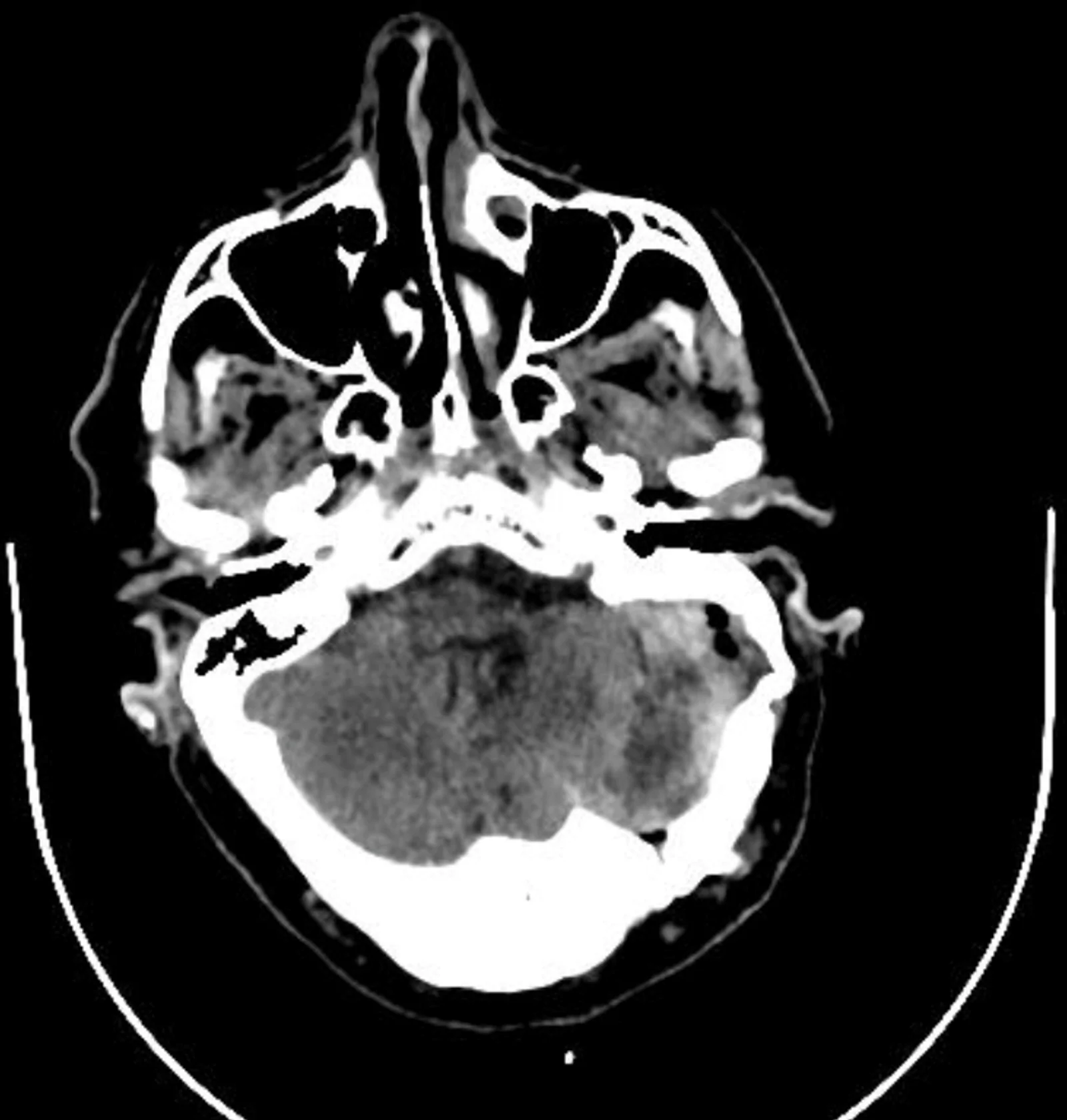
Day 1 postoperative CT scan Postoperative CT scan showing complete removal of the dermoid cyst.

**Figure 6:**
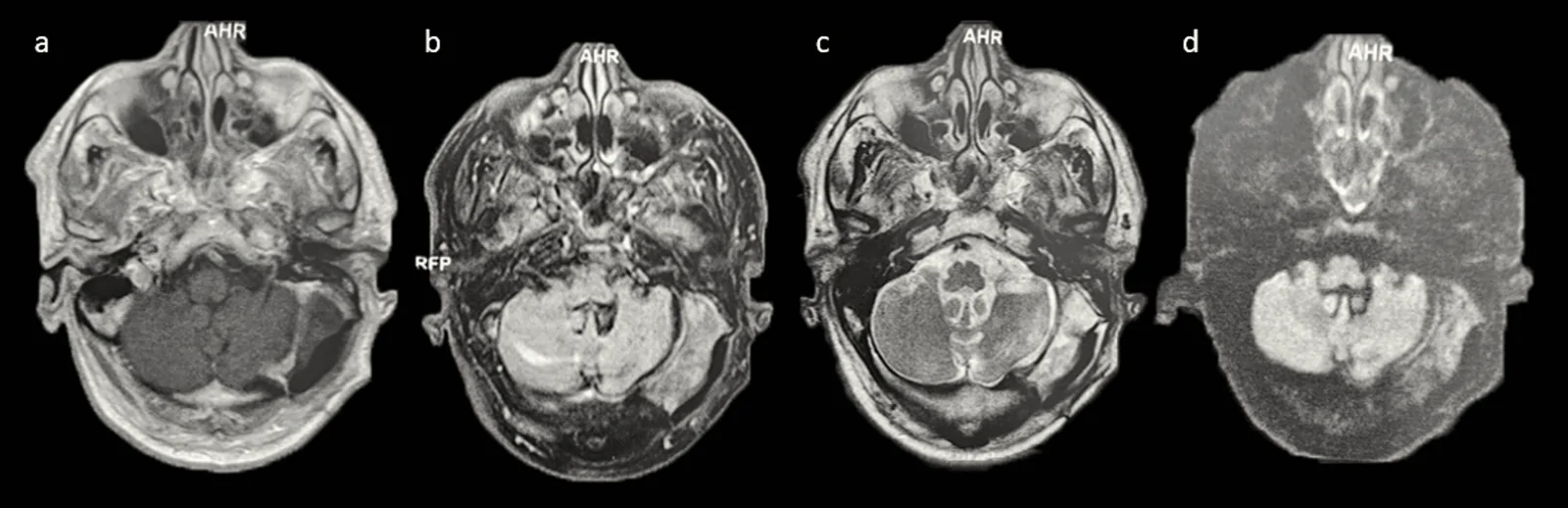
One-year postoperative MRI Axial MRI in T1 WI (a), fluid-attenuated inversion recovery (FLAIR) (b), T2 (c), and diffusion WI (d) showing persistence of surgical cavity in the left cerebellar convexity with no signs of residual tumour nor recurrence.

## Discussion

Dermoid and epidermoid tumours have been described as far back as 1829 by Cruveilhier, when he coined the name "tumeur pérlée" [1,10]. They are rare tumours and account for 0.04-0.7% of all intracranial tumours [1-3], while extradural location accounts for 25% of all cranial DCs [5,6].

Diagnosis is often made in infants and young adults, given the congenital origin of DCs, and they are relatively rare in the elderly population [1,7,8]. In this current case, the patient is 73 years old, which is rather interesting.

DCs are characterized by having both ectodermal and mesodermal components [1,2,4]. Supposedly, they originate from ectopic migration of ectodermal elements within the neural tube during its closure between the third and fifth weeks of embryonic life [1,2,11,12]. This origin would explain the most frequent locations of DCs, such as the midline, in the diploe of the fontanel extradurally, and in the parasellar region intradurally [1,10].

Extradural located DC is an extremely rare occurrence; to our knowledge, only 25 cases are reported in the literature. These cases are summarized in Table 1. These extradural DCs are intradiploic in around 25% of cases [5,6], and they tend to originate from skull sutures located near the skin [12]. Our case is the third described case of DCs in the region of cerebellar convexity, after the cases of Mishra et al. and Esaki et al. [1,13].

**Table 1:** Summary of the described cases of intracranial extradural dermoid cysts

Author and year	Location	Sex	Age (years)
Rubin et al., 1989 [14]	Anterior fontanelle	Female	2
Martinez-Lage et al., 1997 [11]	Posterior fossa	Female	2
	Posterior fossa	Female	6 months
	Posterior fossa	Female	2
Akhaddar et al., 2002 [15]	Posterior Fossa	Female	1
Blythe et al., 2007 [8]	Anterior Fossa	Male	46
Ammirati et al., 2007 [3]	Petrous Apex	Male	49
Sturiale et al., 2009 [16]	Petrous Apex	Female	37
Endo et al., 2011 [7]	Parasellar region	Female	54
Al-Muhaimeed et al., 2012 [17]	Mastoid bone	Female	18
Triplett et al., 2014 [18]	Infratemporal fossa	Female	21
Hashemi et al., 2014 [19]	Occipital area	Female	25
Abderrahmen et al., 2014 [9]	Temporal region	Female	48
Ko et al., 2014 [20]	Lateral sphenoidal ridge	Female	53
Watanabe et al., 2015 [12]	Infratemporal fossa	Female	55
Kim et al., 2017 [21]	Infratemporal fossa	Female	24
Kuran et al., 2017 [22]	Infratemporal fossa	Female	25
Sendjaja et al., 2018 [23]	Infratemporal fossa	Female	43
Özgüral et al., 2018 [6]	Temporal region	Male	38
Hong et al., 2020 [24]	Frontotemporal area	Female	43
Albastaky et al., 2021 [10]	Crista galli	Female	1
Aktham et al., 2023 [25]	Temporal fossa	Female	14
Cao et al., 2023 [2]	Frontotemporal region	Male	58
Mishra et al., 2023 [1]	Cerebellar convexity	Female	34
Pahari et al., 2024 [4]	Region of torcular herophili	Female	14
Current case	Cerebellar convexity	Male	73

As said before, DCs occur mostly in midline locations; lateral locations, such as in our case, are rather rare. Several theories have been presented to explain these locations, although the exact aetiology is still obscure [10,20]. Caldarelli et al. suggested abnormal displacement of primitive ectodermal and mesodermal cells during the cerebral vasculature development along the Virchow-Robin spaces [26], while Martínez-Lage et al. suggested this migration occurs throughout dural invagination through the development of falx cerebri and tentorium cerebelli [6,11]. It has also been proposed that these lateral locations may originate from multipotent embryonic dural cells [1,20]. Other sources suggested the hypothesis of traumatic events resulting in sequestration and inclusion of ectodermal tissue in the development of DCs [1,10].

Clinically, DCs usually have slow-onset manifestations. The wide array of symptoms depends upon the size and location of the tumours, as we can find headache, dizziness, tinnitus, hypoacusis, vision blurring, facial paralysis, facial neuralgia, lower cranial nerve palsy, gait instability, etc. [1,2,9].

Rarely, DCs can rupture in the intracranial space, spontaneously or after trauma [3,12]. The cyst contents will disperse within the intracranial space and provoke an inflammatory response causing ventriculitis, aseptic meningitis, chronic granulomatous arachnoiditis, or even aqueduct stenosis [9,12]. These may lead to seizures or eventually death [1,9]. With early radiological diagnosis, devastating manifestations of DCs could be avoided [10]. Pahari et al. reported a unique case of an infected DC mimicking an abscess [4].

Additionally, DCs can be discovered following the formation of dermal sinuses, which are fistulae connecting the DC with the skin [10,12]. Due to their slow development, DCs are usually quite voluminous at diagnosis. This enlargement is due to accumulation of desquamation products and gland secretion inside the cyst, which means it is not a real neoplasm according to Yaşargil et al. [9,27]. The differential diagnosis of DCs encompasses several different pathologies, such as epidermoid cysts, teratomas, and lipomas, which share a common congenital origin, and other lesions such as arachnoid cysts [10]. Extradural DCs can be confused with skull base lesions, such as chordomas, chondrosarcomas, meningoceles, or metastatic tumours [12]. However, DCs are usually comfortably diagnosed with CT scans and MRI.

On a CT scan, DCs typically appear as a well-circumscribed lesion with hypodense fat content [2,3,12]. Usually, contrast enhancement is absent [2,12]. Meanwhile, the tumour wall frequently presents focal calcifications [2,9]. Characteristically, extradural DCs are associated with skeletal abnormalities, mainly bone erosion [3,6].

On MRI, due to their fatty content, DCs appear with high signal intensity on T1- and T2-weighted images. The low T1 signal in our case is atypical; according to many authors, it may be explained by high amounts of keratin debris and a minimal amount of lipid metabolites within the cyst [28,29]. However, the T2 signal can be heterogeneous, depending on the mixed composition of the tumour, containing variably lipid, cholesterol, and keratin [1,2,9]. Contrast enhancement is usually absent, and proton diffusion on DWI is restricted [2,9]. Fat-suppression techniques are useful to confirm the lipid content of the tumour and thus help with differential diagnosis [9,12]. Extensive enhancment can be seen in the case of ruptured cysts [3]. However, differentiating between extradural and intradural is not obvious radiologically; only bone abnormalities such as erosion could orient toward an extradural lesion [2,20].

In histopathological studies, DCs contain dermal elements, such as hair, follicles, sweat, and sebaceous glands, and they generally have a stratified keratinized squamous epithelial lining [2,9,20].

The consensus in the literature is that maximum safe resection should be the aim of surgical treatment, to maximally reduce the risk of recurrence [1,2,4,12,27]. Sometimes, complete resection is not feasible due to fibrous adhesion of the tumour wall to adjacent structures, which makes aggressive approaches dangerous and results in high morbidity and mortality [12,27].

In some cases, residual epithelial lining on the cyst wall could maintain the process of keratinization and desquamation, and thus be the origin of DC recurrence [2]. Partial resection can also lead to granulomatous reaction or chemical meningitis in case of a dural gap [3,12,27]. Some studies reported the development of squamous cell carcinoma within DC residual remnants [4]. Therefore, regular follow-up is highly recommended.

## Conclusions

This study describes an extremely rare case of an extradural intracranial DC of the left cerebellar convexity discovered in a 73-year-old patient. This case is special because of its extradural and lateral location, as well as its occurrence in an elderly patient. The diagnosis of DCs should be considered in the presence of some characteristic radiological features, although extradural location is not always evident to define. Maximum safe resection should be the aim of surgery to minimize the risk of recurrence and complications.

## References

[1] Mishra MK, Yadav V, Pradhan RS, Prasad RS (2023). Intracranial Interdural and extradural cerebellar convexity dermoid cyst: a case report of rare tumor at the rarest location. Asian J Neurosurg.

[2] Cao D, Xu H, Hu J, Mo J, Yu X, Wang J, Hu H (2023). Frontotemporal giant extradural dermoid cyst: illustrative case. J Neurosurg Case Lessons.

[3] Ammirati M, Delgado M, Slone HW, Ray-Chaudhury A (2007). Extradural dermoid tumor of the petrous apex: case report. J Neurosurg.

[4] Pahari S, Bhandari PB, Sharma M, Baniya P, Devkota D, Jha R, Subedi P (2024). An infected intracranial dermoid cyst at the region of torcular herophili: a case report. Surg Neurol Int.

[5] Nakagawa K, Ohno K, Nojiri T, Hirakawa K (1997). Interdural dermoid cyst of the cavernous sinus presenting with oculomotor palsy: case report. No Shinkei Geka.

[6] Özgüral O, Mammadkhanlı O, Doğan İ, Eroğlu Ü, Heper Okçu A, Ünlü A (20181). Three rare localizations of intracranial dermoid tumors. J Ank Univ Fac Med.

[7] Endo H, Murakami K, Watanabe M, Tominaga T (2011). Extradural dermoid cyst of the parasellar region: a case report. Skull Base Rep.

[8] Blythe JN, Revington PJ, Nelson R (2007). Anterior cranial fossa dermoid cyst: case report. Br J Oral Maxillofac Surg.

[9] Abderrahmen K, Bouhoula A, Aouidj L, Jemel H (2016). Temporal dermoid cyst with unusual imaging appearance: one case report. Turk Neurosurg.

[10] Albastaki A, AlThawadi R, Alajaimi J, Ali KF, Almayman T (2021). An intracranial extradural dermoid cyst presenting with two dermal sinuses and an abscess in a child. Case Rep Neurol Med.

[11] Martínez-Lage JF, Ramos J, Puche A, Poza M (1997). Extradural dermoid tumours of the posterior fossa. Arch Dis Child.

[12] Watanabe K, Filomena CA, Nonaka Y, Matsuda M, Zomorodi AR, Friedman AH, Fukushima T (2015). Extradural dermoid cyst of the anterior infratemporal fossa. Case report. J Neurol Surg Rep.

[13] Esaki T, Harada Y, Nakao Y, Yamamoto T, Mori K (2013). Unusual case of trigeminal neuralgia caused by remote effect of dermoid cyst in the ipsilateral cerebellar hemisphere. Clin Neurol Neurosurg.

[14] Rubin G, Scienza R, Pasqualin A, Rosta L, Da Pian R (1989). Craniocerebral epidermoids and dermoids. A review of 44 cases. Acta Neurochir (Wien).

[15] Akhaddar A, Jiddane M, Chakir N, El Hassani R, Moustarchid B, Bellakhdar F Cerebellar abscesses secondary to occipital dermoid cyst with dermal sinus: case report. Surg Neurol.

[16] Sturiale CL, Mangiola A, Pompucci A, D'Ercole M, Di Muro L, Anile C (2009). Interdural giant dermoid cyst of the petrous apex. J Clin Neurosci.

[17] Al-Muhaimeed HS, Abdelwahed HY, Elgamal EA, Alokby GM, Binnasser AM, Ashraf MM (2012). Extradural dermoid cyst of mastoid bone: a case report. Case Rep Otolaryngol.

[18] Triplett TM, Griffith A, Hatanpaa KJ, Barnett SL (2014). Dermoid cyst of the infratemporal fossa: case report and review of the literature. J Neurol Surg Rep.

[19] Hashemi SM, Hedayat MR, Alghasi M (2014). Intradiploic dermoid cyst: a rare cause of intracranial hypertension. Br J Neurosurg.

[20] Ko SJ, Park KJ, Park DH, Kang SH (2014). Extradural dermoid cyst located in the lateral sphenoid ridge. Brain Tumor Res Treat.

[21] Kim SJ, Baek HJ, Ryu KH (2017). Skull base dermoid cyst in the right infratemporal fossa diagnosed using the Dixon technique: a case report and review of literature. Investig Magn Reson Imaging.

[22] Kuran G, Yazici D (2017). Endoscopic transnasal transpterygoid excision of an infratemporal dermoid cyst. J Craniofac Surg.

[23] Sendjaja AN, Kobayashi H, Morishita T, Abe H, Miki K, Arifin MZ, Inoue T (2018). An infratemporal dermoid cyst that expanded the foramen ovale: a case report. World Neurosurg.

[24] Hong S, Maruyama K, Hatanaka R, Noguchi A, Shimoyamada H, Nagane M, Shiokawa Y (2020). Frontotemporal dermoid cyst with incomplete dermal sinus tract in an adult: a case report. Surg Neurol Int.

[25] Aktham A, Morita S, Takeuchi S (2023). Traumatic rupture of intracranial dermoid cyst with continuous fat droplet migration. Surg Neurol Int.

[26] Caldarelli M, Massimi L, Kondageski C, Di Rocco C (2004). Intracranial midline dermoid and epidermoid cysts in children. J Neurosurg.

[27] Yaşargil MG, Abernathey CD, Sarioglu AC (1989). Microneurosurgical treatment of intracranial dermoid and epidermoid tumors. Neurosurgery.

[28] Wallace D, Tress B, Kwan PF (2008). Radiologically atypical congenital posterior fossa dermoid cyst presenting late in life. J Clin Neurosci.

[29] Orakcioglu B, Halatsch ME, Fortunati M, Unterberg A, Yonekawa Y (2008). Intracranial dermoid cysts: variations of radiological and clinical features. Acta Neurochir (Wien).

